# Statistical learning across cognitive and affective domains: a multidimensional review

**DOI:** 10.3389/fnint.2025.1460471

**Published:** 2025-05-09

**Authors:** Yuyang Wang, Li Lu, Meiyun Wu

**Affiliations:** ^1^Department of Otolaryngology Head and Neck Surgery, Hunan Provincial People's Hospital (First Affiliated Hospital of Hunan Normal University), Changsha, China; ^2^State Key Laboratory of Cognitive Neuroscience and Learning, Beijing Normal University, Beijing, China

**Keywords:** statistical learning, cognitive development, neural mechanism, emotion, predictive coding theory

## Abstract

Statistical learning (SL) is a fundamental cognitive ability enabling individuals to detect and exploit regularities in environmental input. It plays a crucial role in language acquisition, perceptual processing, and social learning, supporting development from infancy through adulthood. In this review, we adopt a multidimensional perspective to synthesize empirical and theoretical findings on SL, covering experimental paradigms, developmental trajectories, and neural mechanisms. Furthermore, we extend the discussion to the emerging intersection between SL and affective processes. Although emotional factors have recently been proposed to modulate SL performance, this area remains underexplored. We highlight current insights and theoretical frameworks addressing the SL–emotion interaction, such as predictive coding theory, and propose directions for future research. This review provides a comprehensive yet focused overview of SL across cognitive and affective domains, aiming to clarify the scope and future potential of this growing field.

## 1 Introduction

Statistical learning (SL) is a foundational cognitive mechanism that enables individuals to extract and utilize regularities from their environment to effectively predict changes. This unconscious process allows the detection and acquisition of statistical patterns across various sensory inputs, facilitating the prediction of future events. SL is widely regarded as a domain-general mechanism that underpins various forms of learning, including language acquisition, music perception, and visual cognition. In this review, SL specifically refers to the ability to discern and predict transitional probabilities and distributional regularities within sequential streams. This capability is particularly critical for navigating dynamic, temporally structured environments. Previous research by Saffran and colleagues demonstrated that even 8-month-old infants could segment linguistic streams based on statistical cues after only brief exposure (Aslin et al., 1998; Saffran et al., 1999). Further studies have revealed that newborns exhibit sensitivity to such statistical patterns, suggesting that SL emerges prenatally and plays a foundational role in early language development (Choi et al., 2020; Kujala et al., 2023). Prosodic features in speech, such as rhythm and intonation, have also been shown to enhance SL by clarifying phrase boundaries (James, 1988). Beyond the auditory-linguistic domain, SL has been demonstrated across various modalities. For example, individuals can acquire visual (Fiser and Aslin, 2002), tactile (Conway and Christiansen, 2005), and musical regularities (Ishida and Nittono, 2023; Jenny et al., 1999), often without conscious awareness. These findings underscore the robustness and flexibility of SL across sensory systems.

In cognitive neuroscience, SL is acknowledged for its pivotal role in comprehending fundamental mechanisms of cognition and behavior. However, recent research has expanded the scope of SL by exploring its interaction with emotional processes. Emotional arousal can modulate attention and enhance the salience of stimuli, thereby influencing learning outcomes (Tyng et al., 2017). This suggests that emotions not only prioritize which environmental cues are processed, but also shape the manner in which these cues are statistically encoded.

To better understand the underlying mechanisms, SL has been situated within predictive processing frameworks. Predictive Coding Theory posits that the brain continuously generates hypotheses about incoming sensory input and updates these predictions to minimize prediction errors (Courville et al., 2006; Friston, 2010; Knill and Pouget, 2004; Koelsch et al., 2019). Within this framework, emotional states may modulate prediction error responses and influence statistical learning outcomes—such as biasing categorical perception under threat-related conditions (Dale et al., 2012; Hasson, 2017; Farmer and Smith, 2014).

This article presents a selective narrative review of statistical learning (SL). Rather than aiming for exhaustive coverage, we focus on representative and influential studies spanning multiple subdomains of SL. Relevant literature was identified through targeted keyword searches in databases such as PubMed and Google Scholar, using search terms related to specific themes (e.g., “statistical learning and development,” “emotional modulation of statistical learning,” “modality differences in statistical learning,” and “neural mechanisms of statistical learning”). Studies were selected based on their relevance, methodological rigor, and theoretical contribution to the field. While this approach allows for a multidimensional synthesis of key findings, we acknowledge that it may not capture the full breadth of research in this rapidly evolving area. This review aims to provide an integrated overview of SL across cognitive and emotional domains. We first introduce core experimental paradigms and examine modality-specific differences. We then discuss developmental trajectories and neural underpinnings, followed by a focused review of the interaction between emotion and SL, highlighting how affective states shape learning processes. Instead of conducting a quantitative meta-analysis, we adopt a thematic and conceptual framework to identify areas of theoretical convergence and highlight key gaps in the literature. Our goal is to offer a comprehensive, multidimensional perspective that bridges traditional cognitive accounts with emerging affective research, and to propose promising directions for future investigation in the field of statistical learning.

## 2 Experimental paradigms in statistical learning

SL has been extensively studied across different modalities such as auditory, visual, and even cross-modal learning. The experimental design of SL tasks typically involves two phases: the learning (familiarization) phase and the testing phase.

**Figure 1:**
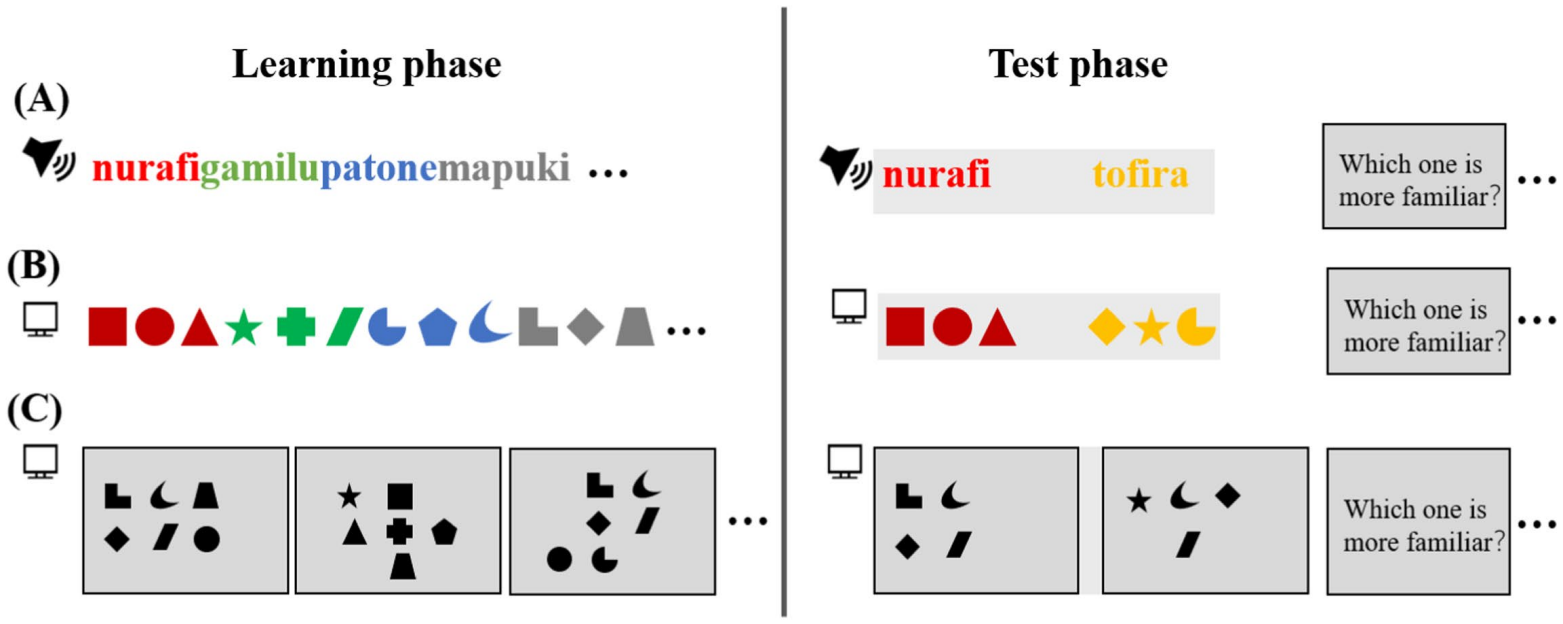
Experimental paradigm. **(A)** The auditory task consists of continuous exposure to four repeating tri-syllabic words followed by a testing phase employing a two-alternative forced choice (2AFC) task. **(B)** The visual task involves a temporal series of four repeating tri-shape sample images followed by a testing phase. **(C)** The visual task includes a spatial arrangement of sub-shape images followed by a testing phase.

During the learning phase (Figure 1), participants are exposed to sequences of stimuli with varying transitional probabilities (TP). High TP between adjacent stimuli indicates a strong association, while low TP suggests weaker or no association. For instance, in an auditory sequence of syllables (e.g., “nu-ra-fi”), the syllables within a word have a TP of 1.0, whereas the TP between different words (e.g., “nurafi” and “gamilu”) is lower (Aslin et al., 1998). Similarly, in visual SL tasks, participants are exposed to sequences of visual shapes, and TP is reflected in the likelihood of one shape following another in a sequence.

Following the learning phase, participants typically undergo a testing phase designed to assess their ability to recognize and group previously encountered patterns. This is often done using tasks such as the two-alternative forced-choice (2AFC), where participants select the more familiar sequence or pattern. Importantly, the design of the testing phase is crucial for accurately assessing SL. It should minimize biases in order presentation and ensure a balance between familiar and novel stimuli.

In addition, SL is influenced by a variety of factors, including individual experience, cognitive impairments, and sensory input channels. These factors collectively shape how individuals perceive and process statistical regularities in their environment.

**Individual Experience**: Performance in statistical learning is influenced by multiple language experience, which significantly impacts infants’ language performance (Kuhl et al., 1992). For instance, individuals experienced in Mandarin demonstrate superior auditory statistical learning of Mandarin compared to those without such experience (Potter et al., 2017). Actually, prior knowledge broadly affects statistical learning (Finn and Hudson Kam, 2008; Toro et al., 2011), which suggests that individual differences prevent a simplistic classification of statistical learning as either “good” or “bad” (Forest et al., 2023).

Prior study has demonstrated that newborns’ neural responses to sounds significantly depend on the context (Todd et al., 2022). Specifically, when common and rare sounds were presented with predetermined probabilities, newborns’ neural responses did not differ, while a notable variance in responses was observed when these sounds alternate with equal probability (Todd et al., 2022), which highlighted the influence of sound sequence structure on statistical learning.

**Cognitive impairment**: Cognitive impairments also affects SL. Children with specific language impairment tend to perform worse on SL tasks compared to their typically developing peers (Obeid et al., 2016), likely due to challenges in predictive coding and semantic processing. Moreover, individuals with developmental dyslexia perform poorly on both linguistic and non- linguistic SL tasks (Pavlidou et al., 2010).

**Sensory deprivation**: Auditory deprivation, particularly early in life, can significantly impair SL abilities (Conway et al., 2011). This deprivation can lead to significant reorganization of cortical functions, affecting how the brain processes sensory information (Kral et al., 2016). However, studies have also indicated that congenitally deaf children with cochlear implants, who are later exposed to auditory stimuli, may retain some auditory SL capabilities (Pesnot Lerousseau et al., 2022), suggesting that exposure to auditory input can mitigate some of the deficits associated with early auditory deprivation. These findings highlight the complex interplay between sensory experiences and cognitive development in shaping SL.

## 3 Developmental patterns of statistical learning

Recent research on statistical learning (SL) has expanded beyond basic pattern recognition to encompass its developmental trajectory, modality-specific characteristics, and the influence of various intrinsic and extrinsic factors. This section explores how SL develops across the lifespan, varies across sensory modalities (auditory, visual, and cross-modal).

### 3.1 Developmental trajectory of statistical learning

SL is not only shaped by experimental design but also by developmental trajectories that vary across individuals and sensory modalities. Auditory SL is present early in life, even in newborns. Infants are able to detect transitional probabilities within syllabic sequences, enabling them to segment words from continuous speech. This ability has been shown to extend to fetuses, suggesting that statistical learning for language may begin before birth (Kujala et al., 2023). Interestingly, while auditory SL in language seems to mature early, other forms of SL, particularly in visual domains, continue to develop throughout childhood, with adults generally outperforming children in visual SL tasks. For instance, Saffran et al. (2006) found no significant differences in auditory statistical learning between six-year-olds and adults, suggesting early maturation in this domain. In contrast, non-linguistic auditory and visual statistical learning abilities tend to improve throughout childhood, with adults generally outperforming children in visual statistical learning tasks (Campbell et al., 2012; Arnon, 2019). In a word, the SL evolves over time also varies across different sensory modalities. Auditory SL has been shown to emerge earlier and develop with more stability compared to visual SL, which typically requires more experience and exposure to reach optimal performance. This suggests a possible innate predisposition for processing linguistic information, though the lack of developmental change in auditory-syllable statistical learning remains intriguing.

Notably, measurement methods also play a critical role in capturing developmental changes in SL. As reviewed by Forest et al. (2023), direct measures (e.g., two-alternative forced-choice tasks) assess recognition of learned sequences, while indirect measures (e.g., reaction times or neural responses) can detect learning that occurs implicitly during exposure (Fiser and Aslin, 2001; Forest et al., 2019; Jacoby, 1991; Schlichting et al., 2017). The choice of method may affect the interpretation of developmental patterns and learning efficacy.

### 3.2 Modality-specific constraints in statistical learning

Extensive empirical evidence indicates that statistical learning (SL) is modality-specific (Frost et al., 2015; Walk and Conway, 2016). Individuals can simultaneously learn statistical regularities in different sensory modalities without interference (Conway and Christiansen, 2006; Mitchel and Weiss, 2011). Additionally, significant performance differences have been observed across various sensory modalities (Emberson et al., 2011; Kemény and Lukács, 2019; Qi et al., 2018). Auditory statistical learning tends to develop earlier and more rapidly than visual statistical learning, highlighting distinct characteristics and impacts across modalities (Emberson et al., 2019). Although both rely on transitional probabilities during the learning phase, a study found a relatively low correlation between performance in visual and auditory modalities (Siegelman and Frost, 2015).

**Auditory SL**: Most auditory statistical learning studies utilize a paradigm developed by Saffran et al. (Aslin et al., 1998; Saffran et al., 1999). Early research demonstrated that infants can track syllable probabilities in continuous speech. These findings have been interpreted as evidence that learning mechanisms may rely on predictions about both timing and content (Wilson, 2003). Neural imaging studies showed that the left posterior temporal gyrus responds to embedded speech regularities in auditory linguistic statistical learning but not in non-linguistic contexts (Schneider et al., 2024). Using multivoxel pattern similarity analysis, researchers identified similarities between the neural representations of auditory linguistic statistical learning and language processing, specifically within the left posterior temporal gyrus (Schneider et al., 2024).

**Visual SL**: Kirkham et al. (2002) conducted visual statistical learning tasks with infants aged 2, 5, and 8 months and found no significant differences in learning performance among these ages. However, 11-month-old infants successfully learned visual regularities, indicating an age-related increase in capability (Natasha Z. Kirkham et al., 2007). Further studies show that 16-month-old infants struggle with recognizing altered facial images, suggesting basic processing capabilities (Antovich et al., 2020). A study involving 183 children aged 5 to 12 years revealed that longer visual stimulus durations correlated with improved performance as age increased, highlighting the influence of age and stimulus duration on statistical learning (Arciuli and Simpson, 2011).

**Cross-modality SL**: Our environment offers structured information across sensory modalities, with statistical learning influenced by cross-modal cues that can either enhance or inhibit learning. Learners can extract multiple statistical regularities when cross-modal coherence is present. Contrary to prior models, statistical learning in one modality is not independent of other modalities, especially when cross-modal relationships are inconsistent. The McGurk illusion, for example, supports learning of transitional probabilities between audio-visual inconsistent yet integrated percept (Mitchel et al., 2014). Research demonstrates that statistical learning can be enhanced or reduced based on cues from a second modality (Duato et al., 2023; Glicksohn and Cohen, 2013; Mitchel and Weiss, 2011).

Studies comparing visual and auditory language learning in children aged 5 to 12 years show that visual learning improves with age, while auditory performance remains stable (Raviv and Arnon, 2018). In adults, auditory statistical learning improves significantly when accompanied by visual stimuli, whereas infants show no significant differences (Thiessen, 2010), highlighting developmental differences between auditory language and vision (Moreau et al., 2022; Saffran et al., 2006). fMRI studies reveal increased activity in the superior temporal sulcus and frontal cortex when processing linked stimuli across different modalities, indicating these regions’ importance in cross-modal statistical learning (Frost and Monaghan, 2016). Comprehensive explanations of multisensory statistical learning are necessary to fully understand these processes.

## 4 Neural mechanisms in statistical learning

The neural mechanisms underlying SL involved in SL spans both temporal dynamics, as revealed by electroencephalography (EEG), and spatial patterns, as investigated using functional magnetic resonance imaging (fMRI). This discussion will examine the evidence for each of these aspects, delving into their distinct contributions to our understanding of SL processes. By analyzing these dimensions separately, we can better appreciate the complex interplay between the timing of neural activity and the spatial localization of brain regions involved in SL.

### 4.1 Dynamic temporal dimension

Recent EEG studies have highlighted the importance of neural entrainment in predicting statistical learning performance (Choi et al., 2020). Neural entrainment involves the alignment of brain oscillations to the rhythmic patterns of external stimuli, thereby synchronizing neural activity with cognitive processes (Calderone et al., 2014). During the learning phase, repeated exposure to stimuli enhances neural entrainment, and the strength of this activity predicts the ability to differentiate between familiar and novel words (Choi et al., 2020).

A previous study has shown that both full-term and preterm infants exhibit neural entrainment to syllable and word frequencies in continuous speech stimuli (Kabdebon et al., 2015). Furthermore, infants demonstrate learning capabilities during sleep, with increased power and phase-locking values (PLV) in the occipital and temporal lobes for word frequencies (Benjamin et al., 2023; Fló et al., 2019). Event-related potential (ERP) studies consistently reveal that newborns can recognize word boundaries using prosodic cues, with electrodes in the frontal, temporal, and parietal regions of the right hemisphere showing higher responses compared to those in the left hemisphere (Teinonen et al., 2009). Interestingly, despite differences in experience, the neural responses to syllables in infants and adults are remarkably similar (Choi et al., 2020).

Additionally, words elicit a larger N400 effect compared to non-words during the learning phase, emerging rapidly after just 1 min of exposure (Cunillera et al., 2009). The N400 component, characterized by a negative deflection around 400 milliseconds post-stimulus, is extensively used to investigate language processing and semantic memory (Kutas and Hillyard, 1980). Balaguer et al. also confirmed that the N400 amplitude increases with exposure time, with greater effects observed in the second minute compared to the first, indicating dynamic changes in neural activity during statistical learning (De Diego-Balaguer et al., 2007). These findings suggest that neonates possess inherent auditory capabilities for language learning, including the ability to segment speech, detect word boundaries, learn words, and extract prosodic cues. Moreover, intracranial EEG studies show that early stages of processing involve lower-level features, while higher-level units are handled in later stages (Henin et al., 2020).

### 4.2 Spatial dimension

In adults, non-linguistic auditory statistical learning studied via magnetoencephalography (MEG) revealed increased neural synchrony under structured conditions (e.g., 12 tones divided into four three-tone combinations) compared to random conditions (e.g., 12 tones presented randomly). Source analysis indicated that neural synchrony in the left central frontal gyrus and right temporo-frontal area could predict behavioral performance (Moser et al., 2021). In addition, neural activity in the inferior frontal gyrus during structured sequence exposure was higher than during random sequence exposure, observed in both adults and children (Karuza et al., 2013; McNealy et al., 2010). The involvement of the inferior frontal gyrus suggests sensitivity to structured sequences, reflecting specific transitions in stimulus presentation (Schapiro et al., 2013).

Humans typically segment continuous sequences into smaller units during processing, such as words and events. Previous fMRI studies have demonstrated significant activation of the left inferior frontal gyrus, which is associated with the ability to differentiate between sequences with high statistical coherence (e.g., words) and lower coherence (e.g., part-words; Karuza et al., 2013). Activation in this region increases with greater exposure to items from the same group, indicating sensitivity to specific relationships within structured streams, similar to transitions observed in visual statistical learning (Schapiro et al., 2013).

Early fMRI studies identified significant activation in the bilateral superior and middle temporal gyri during language word segmentation tasks (McNealy et al., 2006). Compared to adults, children exhibited stronger neural responses in these areas (McNealy et al., 2010), indicating that children were more sensitive to statistical learning. This is consistent with early findings that infants were sensitive to word units (Teinonen et al., 2009), suggesting children exhibit a higher sensitivity in words. Additionally, cortical activity in the temporal lobe shows pronounced left lateralization when processing sounds containing statistical and speech cues (McNealy et al., 2006). The striatum was also activated during word segmentation tasks (Seger and Spiering, 2011), a response observed not only in the auditory domain (Karuza et al., 2013; McNealy et al., 2006) but also in the visual domain (Turk-Browne et al., 2009).

**Figure 2:**
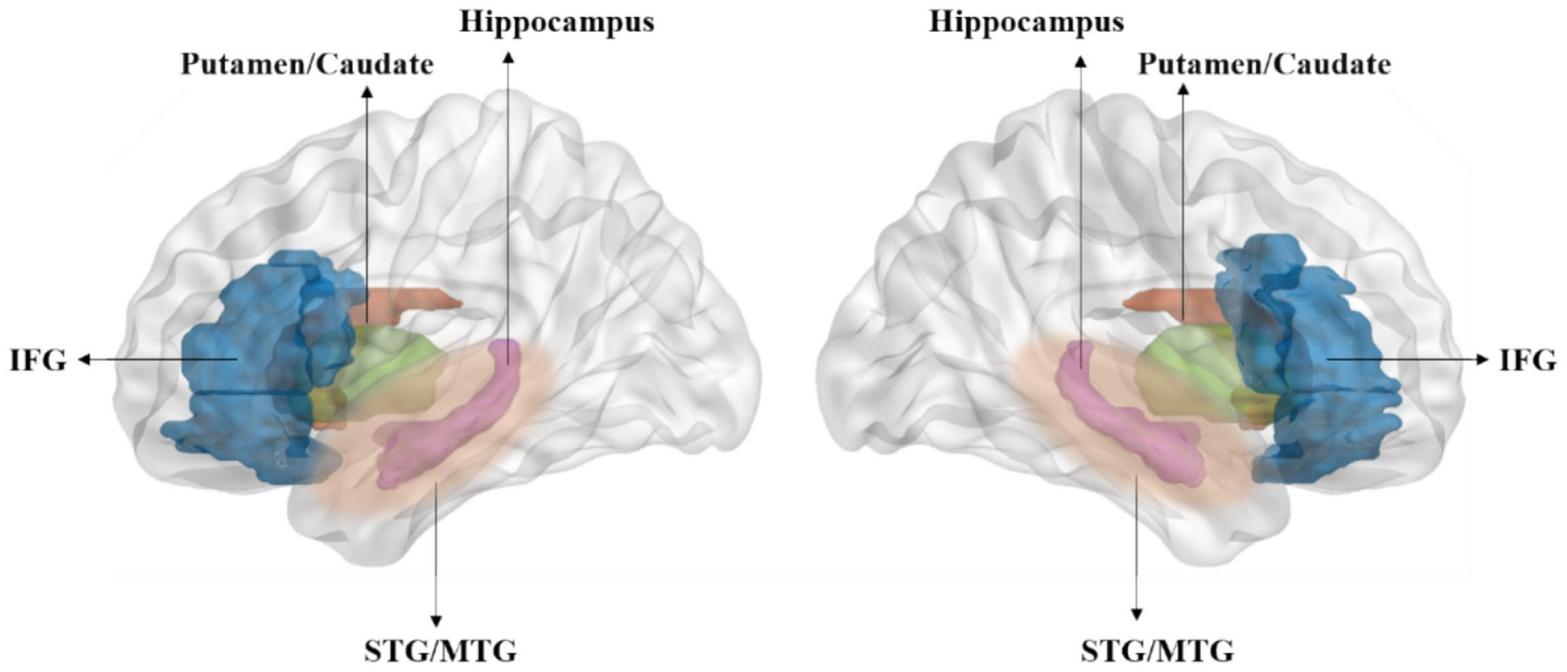
Statistical learning involves several key brain regions, including the hippocampus, inferior frontal gyrus (IFG), superior and middle temporal gyrus (STG/MTG), and the putamen and caudate within the temporal lobe (Karuza et al., 2013; Frost et al., 2015; Mcnealy et al., 2006; Turk-Browne et al., 2009).

Neuroimaging studies on children reveal that auditory statistical learning involves cooperation between the superior temporal gyrus and frontal lobe areas (Figure 2), a pattern also observed in early childhood stages (Finn et al., 2019; McNealy et al., 2010; Schlichting et al., 2017). The superior temporal gyrus plays an integrative role in speech perception (Bhaya-Grossman and Chang, 2022). Furthermore, research indicates a close correlation between statistical learning performance and activation of the left arcuate fasciculus (López-Barroso et al., 2013). Numerous memory system areas, including the putamen and caudate in the temporal lobe, are also involved in statistical learning (Karuza et al., 2013; Frost et al., 2015).

Recent studies indicate that the hippocampus played a pivotal role in word segmentation tasks (Finn et al., 2019; Schlichting et al., 2017). An intracranial EEG study on seven patients with intractable epilepsy, exposed to a stream of 24 trisyllabic speech stimuli, found that while the auditory cortex primarily responds to syllable frequency, the hippocampus generates responses to word frequency, underscoring its significant role in speech segmentation processing (Ramos-Escobar et al., 2022). This suggests a hierarchical relationship between the auditory cortex and hippocampus in speech processing. Consistently, previous research has shown that individuals with hippocampal impairment perform worse in visual statistical learning tasks (Cerreta et al., 2018), highlighting the critical role of the hippocampus in such learning tasks. Moreover, the cortical thickness of the left inferior frontal gyrus and the volume of the right hippocampus have been shown to predict statistical learning performance, particularly in older children (Finn et al., 2019). Accordingly, animal model studies have demonstrated that damaging one system could improve performance on tasks dependent on another system, revealing a possible competitive mechanism between the hippocampus and striatal memory systems (Poldrack and Packard, 2003). These findings collectively indicate the complex role of the hippocampus in statistical learning and language processing, and its interactions with other brain regions, such as the inferior frontal gyrus.

Independent component analysis of MRI data indicates that statistical learning generally activates auditory and premotor area networks. In some individuals, high auditory-motor synchrony selectively activates the frontoparietal network, correlating positively with improved learning performance (Orpella et al., 2022). These studies highlight the complexity of statistical learning in cognitive processing and neural mechanisms, as well as the specific roles of different neural networks during the learning process.

## 5 Emotion in statistical learning

Previous studies on statistical learning (SL) have primarily focused on its cognitive aspects, considering it as a fundamental mechanism in human cognition (Bogaerts et al., 2020; Sherman et al., 2020). However, the role of emotion in statistical learning—both in how emotions are developed through SL and how SL is modulated by emotions—is critical for a comprehensive understanding of the human brain. Recent research has begun to explore these questions, though they remain under-examined. Understanding the interaction between emotions and SL is essential for several reasons.

Firstly, emotions fundamentally shape human experiences and behaviors. Investigating how SL influences emotional development can provide insights into the mechanisms by which individuals learn emotional responses from their environments. Secondly, exploring how emotions modulate SL can reveal how emotional states impact the ability to detect and predict patterns, crucial for adaptive behavior and decision-making. This dual perspective enhances our understanding of the interplay between cognition and emotion, potentially leading to applications in improving learning strategies, therapeutic interventions for emotional disorders, and the design of emotionally intelligent artificial intelligence systems. Consequently, integrating the study of emotions with SL is imperative for a holistic understanding of the human brain and its complex functionalities. Then, the following sections will review related empirical research and propose a tentative explanatory framework using Predictive Coding (PC) Theory to reveal how SL and emotion interact.

**Figure 3:**
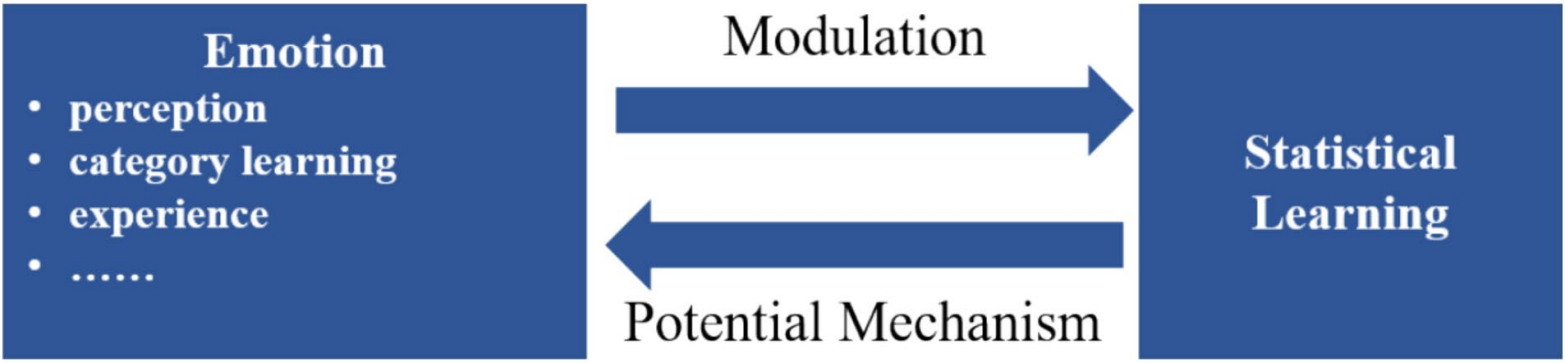
Emotion-SL interactions.

The interaction between statistical learning (SL) and emotion manifests in two primary aspects (Figure 3). Firstly, emotional development may utilize SL mechanisms. Recent studies suggest a close intertwining of language and emotions, where the acquisition of emotional categories parallels language acquisition processes (Ruba et al., 2022). The formation of emotional categories results from the interplay of genetic and environmental factors, contributing to the ongoing nature versus nurture debate (Cowen and Keltner, 2021). As a cross-domain mechanism, SL applies similarly to the domain of emotions (Frost et al., 2015). Secondly, the interaction of emotion and cognitive processing reveals that emotions can influence and regulate cognitive processes (Dreisbach, 2022; Todd et al., 2020; Tyng et al., 2017). This interaction may introduce biases in information processing during SL, with emotions potentially modulating this process. The emotion-SL interaction spans various fields, including emotional development, musical psychology, social cognitive processing, and clinical psychology.

### 5.1 Emotional statistical learning

In terms of emotional development, emotion perception, emotion category learning, and emotion experience are acquired and developed through SL. While some argue that humans have universal, evolutionarily constant basic emotions (Ekman, 1992), children’s emotional perception, understanding, and behavior are shaped by their diverse emotional environments over time (LoBue and Ogren, 2022). The statistical patterns in the socioemotional inputs children encounter influence their emotional learning by enabling them to discern regularities in their social environment (Plate et al., 2022b). SL is likely a mechanism for emotional learning, allowing learners to sift through extensive and intricate inputs to identify patterns and regularities in the social world.

Developmentally, SL has been observed to emerge in infants—even neonates—and follows different trajectories from infancy to adulthood for direct and indirect measures (for a review, see Forest et al., 2023). Indirect measures, such as reaction time, saccade latencies, and neural responses, suggest minimal changes in SL across development. In contrast, direct measures, such as performance on two-alternative forced-choice (2AFC) post-tests, demonstrate improved SL for auditory non-linguistic and visual inputs from young children to adults.

Milestones in emotional development illustrate the role of SL in shaping emotion concepts. By 6 months, infants can discriminate between different facial expressions, such as anger, sadness, fear, and happiness (Haviland and Lelwica, 1987; Hoehl and Striano, 2008; Izard et al., 2010). By ages two to three, young children begin to show distinct emotion concepts, and older children aged four to nine expand these concepts to include fear, surprise, and disgust (Widen et al., 2015). Emotion concepts refine throughout development, and large-scale MRI data involving children aged 5 to 15 years for emotional encoding suggest that emotion concept representations become relatively stable by mid to late childhood, with synchronization observed during adolescence (Camacho et al., 2023). These findings underscore the intrinsic connection between SL and emotional development.

Existing studies have used traditional SL paradigms to investigate emotional perception and category learning across different age groups, including facial expressions, tone of voice, and body language. These studies demonstrate that individuals acquire emotional cues from their surroundings using frequency distributions and transitional probabilities to infer and predict behavior (Plate et al., 2022b; Ruba et al., 2022). For instance, research has shown that 12-month-old infants can extract statistical information from sequences of emotional faces, indicating their capacity to discern differences in transitional probabilities within streams of emotional stimuli (Mermier et al., 2022), foundational for early social interactions. Subsequent studies support this finding, illustrating infants’ ability to learn dynamic patterns of emotional transitions from caregivers (Nencheva et al., 2024). Plate et al. (2019) found that children (aged 6 to 8 years) and adults (aged 18 to 22 years) rapidly changed their boundary judgments of facial expression categories after exposure to facial expressions with different statistical distributions. This supports the hypothesis that SL underpins the formation of emotion categories (Woodard et al., 2021, 2022). Learners demonstrate flexibility in updating emotional category boundaries to adapt to environmental experiences. For instance, children aged 6 to 12 years exhibited more flexible SL abilities for emotional information compared to other types of information (Plate et al., 2023). Similarly, adults display differential responses in SL to visual stimuli based on emotional valence, with emotional cues enhancing sensitivity to transitional probabilities relative to neutral stimuli (Everaert et al., 2020).

Furthermore, the experience of listening to music, often linked to SL and probabilistic prediction processes, demonstrates the emotional impact of SL (Koelsch et al., 2019; Pearce, 2018). For example, music-evoked pleasure is predicted jointly by uncertainty and surprise (Cheung et al., 2019). This view is supported by numerous brain imaging and computational modeling studies (Daikoku, 2019, 2023; Pearce et al., 2010; Stupacher et al., 2022), suggesting that emotional experiences, such as musical esthetics, can be shaped by SL.

### 5.2 Statistical learning modulated by emotion

The interaction between emotions and cognition illustrates how emotions influence our information processing (Dreisbach, 2022; Todd et al., 2020; Tyng et al., 2017). From an evolutionary perspective, emotions, particularly negative ones like fear or anger, were prioritized by the brain due to their survival importance. Studies show that the fusiform gyrus exhibits heightened neural responses to fearful expressions (Morris et al., 1998; Surguladze et al., 2003), a phenomenon potentially modulated by feedback from the amygdala (Vuilleumier et al., 2004). This highlights the neural mechanisms for allocating resources in response to emotional stimuli, which are further influenced by genetic factors, individual personality traits, and anxiety levels (Patrik Vuilleumier, 2005).

The impact of emotions on SL, a crucial cognitive mechanism for deciphering environmental regularities, is significant but not yet fully understood. For instance, Kverno (2000) found that anxiety traits influence individuals’ frequency estimates and performance in recall tasks for neutral and threatening words. Individuals with high anxiety traits focused more on threatening words during frequency estimation and made more errors in false recognition tasks, indicating emotional influences on SL.

The differential impact of emotional versus non-emotional stimuli on statistical learning has emerged as a significant area of study. Research has demonstrated that SL outcomes are superior with emotional stimuli compared to non-emotional ones (Plate et al., 2022a). These learning effects are more pronounced with consistent emotional cues, underscoring the role of emotional information in social interactions. In clinical psychology, researchers have explored the potential applications of SL in diagnosing and intervening in mental disorders and developmental disabilities (Saffran, 2018). Studies indicate that children with autism spectrum disorder (ASD) often exhibit poorer performance in SL tasks compared to typically developing children (Jones et al., 2018). Adults with high autism traits excelled in tasks with non-social stimuli compared to those with low traits, but only the latter showed significant learning with socially meaningful stimuli (Li et al., 2023). Recent reports suggest that autism symptoms may arise from impaired predictive processes, with reduced connectivity in neural networks, including those linking the prefrontal cortex and sensory areas, contributing to deficits in predictive processing observed in ASD (Lawson et al., 2014; Pellicano and Burr, 2012). Atypical sensory processing in ASD may result from an inability to modulate sensory input based on prior expectations, leading to sensory overload or distress (Sinha et al., 2014). However, recent research in adult populations challenged these findings, indicating intact predictive processing in autistic adults (Pesthy et al., 2023). Future research is needed to elucidate the origins of these discrepancies.

A study on patients with social anxiety disorder undergoing attention bias modification (ABM) therapy found that their SL ability could predict therapeutic outcomes (Alon et al., 2019). Another study explored the relationship between negative emotional symptoms (such as depression, anxiety, and stress) and cognitive biases in information processing, showing that emotional states influenced both SL and cognitive biases (Herff et al., 2023). These findings highlight the critical role of emotional modulation in SL, enhancing our understanding of the interplay among emotion, cognition, and learning, with potential implications for psychological interventions and therapies.

### 5.3 Predictive coding theory in emotion-statistical learning interaction

Predictive processing is crucial for SL (Dale et al., 2012; Hasson, 2017). Online measures of SL suggest that participants make implicit predictions as they learn (Farmer and Smith, 2014). Predictive Coding (PC) Theory offers a framework for understanding how the brain processes information, acquires emotion categories, and makes inferences (Barrett, 2017; Smith et al., 2019; Wager et al., 2015). Initially proposed by Friston, this theory integrates empirical Bayes theory and a hierarchical model of cortical processing, suggesting that the brain predicts sensory inputs and adjusts these predictions based on actual sensory data to minimize prediction errors (Arnal and Giraud, 2012; Friston, 2005, 2010; Friston and Kiebel, 2009; Huang and Rao, 2011). This process enables efficient responses to environmental stimuli (Courville et al., 2006; Friston, 2010; Knill and Pouget, 2004; Spratling, 2017).

**Figure 4:**
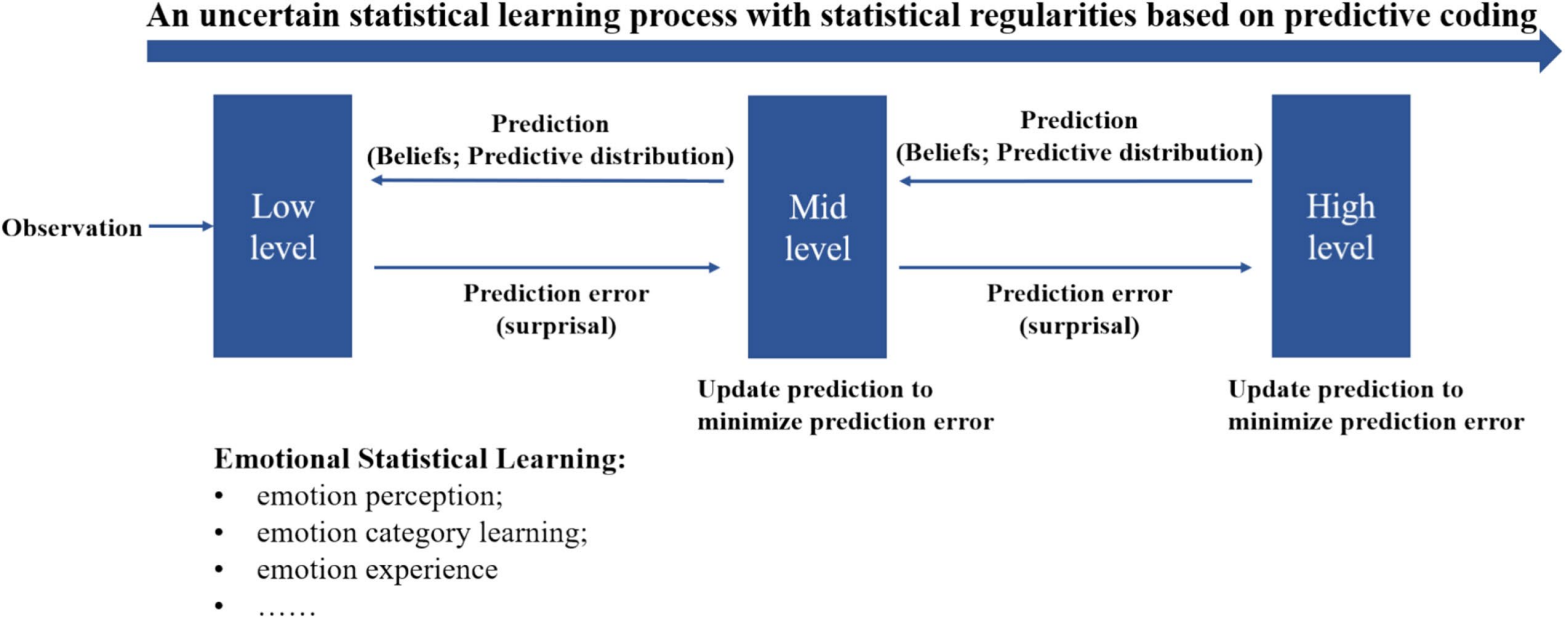
Predictive coding theory on statistical learning (emotion SL as a specific case).

According to PC theory, the brain functions as a hierarchically organized system where higher processing levels attempt to predict the potential causes of sensory inputs received from lower levels. Neurons in higher layers generate predictions about incoming signals, continuously compared with actual signals from lower layers. This comparison allows the brain to reinforce or update its predictions based on whether they match the incoming signals. When predictions are incorrect, a prediction error signal is sent back to the predictive neurons, prompting adjustments. These recursive loops of predictions and error signals enable the brain to maintain accurate and up-to-date representations of both internal states and external stimuli. The concept of prediction error, a core element of PC Theory, has been applied to explore how individuals process and predict emotional and uncertainty information in social interactions, including decision-making and impression management behaviors (FeldmanHall and Shenhav, 2019; Heffner and FeldmanHall, 2022; Heffner et al., 2021). This theory provides a framework for understanding the interaction between emotion and SL (Figure 4). Emotional statistical learning is a specific case of SL that can be implemented by predictive processing. The beliefs and predictive distributions during predictive processing dynamically shape human emotional experience and understanding.

**Figure 5:**
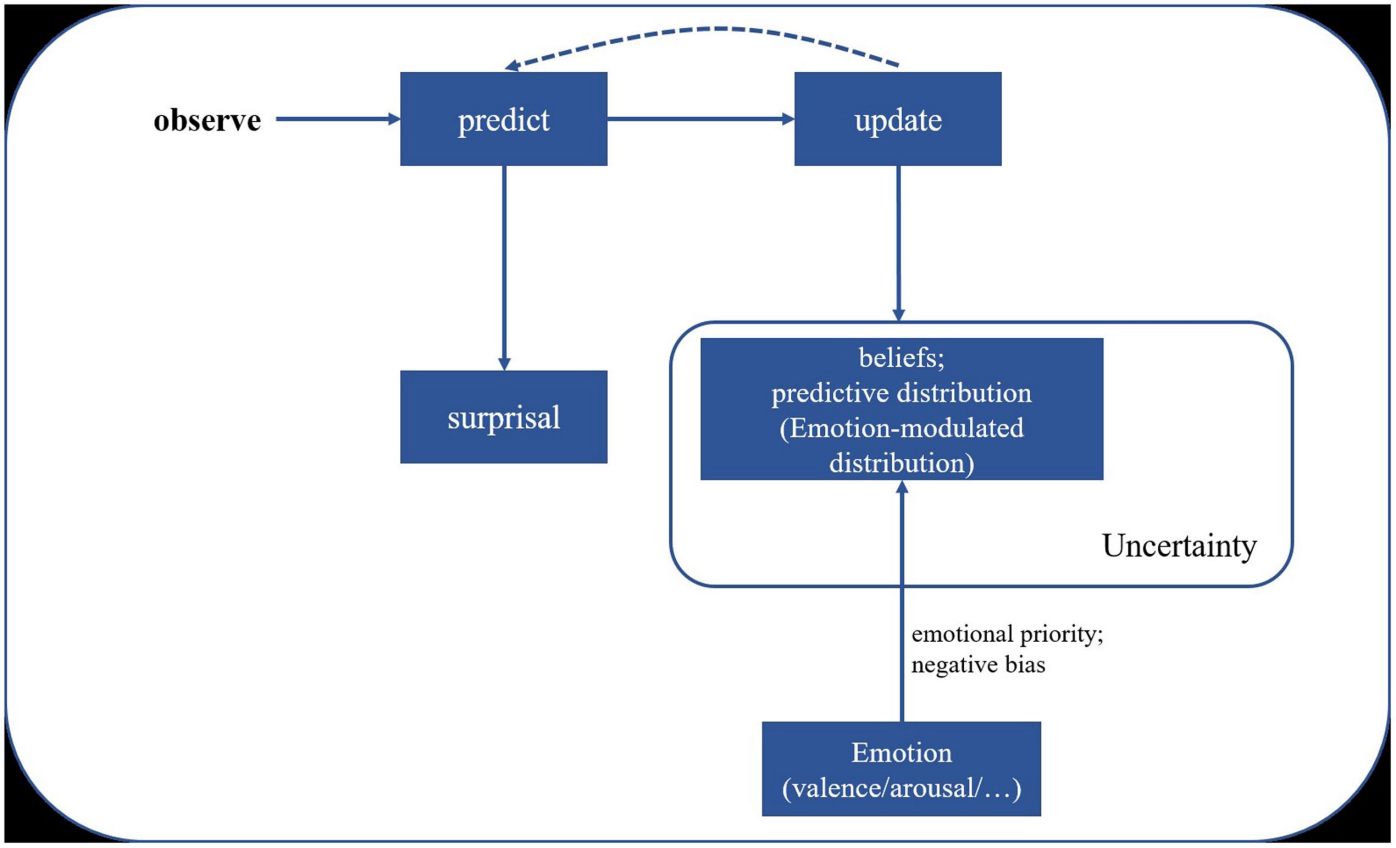
Emotion-modulated statistical learning based on predictive coding theory. Adapted from the computational framework for investigating predictive processing proposed by Skerritt-Davis and Elhilali (2021).

To illustrate the modulation effect of emotions on statistical learning, we propose a tentative framework (Figure 5) base on the computational framework for investigating predictive processing developed by Skerritt-Davis and Elhilali (2021). This model maintains a set of context beliefs or hypotheses representing previous input contexts, which are assumed to be inherently modulated by emotional factors such as emotional priority and negative bias (e.g., prioritizing emotional information and weighting more on negative emotional stimuli). This results in an emotion-modulated predictive distribution during processing. However, further validation of this interpretative framework is necessary through future empirical studies.

Bayesian computational models and theories of constructed emotion, aligned with neural computation and evolutionary biology principles, provide a novel perspective on the neural mechanisms underlying emotion (Barrett, 2017). The role of cross-modal social signals, such as facial expressions and vocal cues, in social cognition underscores the relevance of SL mechanisms. These mechanisms are pivotal in understanding emotion-related interpersonal communication and social cognition (Turesson and Ghazanfar, 2011). Researchers have developed computational models of implicit emotional learning based on prediction errors and statistical inference (Puviani and Rama, 2016; Puviani et al., 2021). Additionally, mathematical models integrating emotional valence and arousal, grounded in the free energy theory, hold promise for applications in emotional intervention and computational approaches (Usuda and Yanagisawa, 2022).

## 6 Summary and open questions

SL research encompasses a broad range of domains, from behavioral studies to neural investigations, exploring its manifestations across auditory, visual, and tactile modalities and among diverse age groups. A notable trend is the increasing focus on how emotions influence SL, which promises to deepen our understanding of both cognitive and emotional processing.

Advancements in brain imaging technologies provide new avenues for examining the neural mechanisms underlying SL with both high temporal and spatial resolution. Future studies could explore how information is distributed across the brain and the effects of cross-modal integration, such as the interaction between auditory and visual information, on SL. Neuroimaging studies have identified key areas involved in SL tasks, including the hippocampus, inferior frontal gyrus, striatum, and superior temporal gyrus. Further exploration is needed to understand how these brain regions cooperate in the SL process.

In conclusion, SL is a fundamental cognitive mechanism with significant implications across developmental stages, neural foundations, individual differences, and states of consciousness. By elucidating the neural mechanisms and emotional modulation of SL, we can achieve a more comprehensive understanding of human learning processes. This understanding not only clarifies the complexities of cognitive development but also offers important theoretical insights for enhancing learning efficiency and developing targeted interventions. Future research should continue to explore these dimensions to fully grasp the intricate relationships between emotion, learning, and brain function.
